# ERβ1: characterization, prognosis, and evaluation of treatment strategies in ERα-positive and -negative breast cancer

**DOI:** 10.1186/1471-2407-14-749

**Published:** 2014-10-07

**Authors:** Jordan M Reese, Vera J Suman, Malayannan Subramaniam, Xianglin Wu, Vivian Negron, Anne Gingery, Kevin S Pitel, Sejal S Shah, Heather E Cunliffe, Ann E McCullough, Barbara A Pockaj, Fergus J Couch, Janet E Olson, Carol Reynolds, Wilma L Lingle, Thomas C Spelsberg, Matthew P Goetz, James N Ingle, John R Hawse

**Affiliations:** Department of Biochemistry and Molecular Biology, Mayo Clinic, 16-01B Guggenheim Building, 200 First St. SW, Rochester, MN 55905 USA; Department of Molecular Pharmacology and Experimental Therapeutics, Mayo Clinic, Rochester, MN USA; Department of Biomedical Statistics and Informatics, Mayo Clinic, Rochester, MN USA; Department of Laboratory Medicine and Pathology, Mayo Clinic, Rochester, MN USA; The Department of Pathology, Dunedin School of Medicine, University of Otago, Dunedin, New Zealand; Department of Laboratory Medicine and Pathology, Mayo Clinic, Scottsdale, AZ USA; Department of Surgery, Mayo Clinic, Scottsdale, AZ USA; Department of Health Sciences Research, Mayo Clinic, Rochester, MN USA; Department of Oncology, Mayo Clinic, Rochester, MN USA

**Keywords:** Estrogen receptor beta, Breast cancer, Estrogen receptor alpha, Triple negative breast cancer, Therapy

## Abstract

**Background:**

The role and clinical value of ERβ1 expression is controversial and recent data demonstrates that many ERβ antibodies are insensitive and/or non-specific. Therefore, we sought to comprehensively characterize ERβ1 expression across all sub-types of breast cancer using a validated antibody and determine the roles of this receptor in mediating response to multiple forms of endocrine therapy both in the presence and absence of ERα expression.

**Methods:**

Nuclear and cytoplasmic expression patterns of ERβ1 were analyzed in three patient cohorts, including a retrospective analysis of a prospective adjuvant tamoxifen study and a triple negative breast cancer cohort. To investigate the utility of therapeutically targeting ERβ1, we generated multiple ERβ1 expressing cell model systems and determined their proliferative responses following anti-estrogenic or ERβ-specific agonist exposure.

**Results:**

Nuclear ERβ1 was shown to be expressed across all major sub-types of breast cancer, including 25% of triple negative breast cancers and 33% of ER-positive tumors, and was associated with significantly improved outcomes in ERα-positive tamoxifen-treated patients. In agreement with these observations, ERβ1 expression sensitized ERα-positive breast cancer cells to the anti-cancer effects of selective estrogen receptor modulators (SERMs). However, in the absence of ERα expression, ERβ-specific agonists potently inhibited cell proliferation rates while anti-estrogenic therapies were ineffective.

**Conclusions:**

Using a validated antibody, we have confirmed that nuclear ERβ1 expression is commonly present in breast cancer and is prognostic in tamoxifen-treated patients. Using multiple breast cancer cell lines, ERβ appears to be a novel therapeutic target. However, the efficacy of SERMs and ERβ-specific agonists differ as a function of ERα expression.

**Electronic supplementary material:**

The online version of this article (doi:10.1186/1471-2407-14-749) contains supplementary material, which is available to authorized users.

## Background

The global incidence of breast cancer has grown from 1980 to 2010 at an annual rate of 3.1%. In 2010, there were 1.65 million women diagnosed with breast cancer and 425,000 deaths caused by this disease [1]. Despite the substantial advances in understanding breast cancer biology, the clinical management of women with this disease continues to rely almost solely on the tumoral expression of estrogen receptor alpha (ERα), progesterone receptor (PR) and epidermal growth factor receptor 2 (HER2). ERα is expressed in approximately 70% of all breast tumors and is the basis for the use of selective estrogen receptor modulators (SERMs) and aromatase inhibitors (AIs), which substantially reduce the risk for disease recurrence and prolong patient survival. Despite the discovery of a second form of the ER, ERβ1, more than 15 years ago [2, 3], the endocrine sensitivity and ER status of breast tumors continues to be clinically defined exclusively by ERα expression [4–6].

Like ERα, ERβ1 is a member of the nuclear receptor superfamily of proteins that functions as a ligand-mediated transcription factor [3]. The DNA binding domains of ERα and ERβ1 share 96% homology at the amino acid level, however, the remainder of the protein domains are highly divergent with the hinge region, AF1 domain, and ligand binding domain sharing only 30%, 30% and 53% conservation respectively [3, 7]. A number of microarray studies from our laboratory and others have demonstrated that these two proteins function differently in response to both estrogen and anti-estrogens [8–14]. Consistent with these data, the genome wide chromatin binding profiles, or cistromes, of ERα and ERβ1 share only 40% overlap following short term estrogen treatment [14].

While ERβ is highly expressed in normal breast tissue [15–21], a number of immunohistochemistry-based studies have demonstrated conflicting data with regard to ERβ expression in breast tumors. For example, the frequency of ERβ expression in breast tumors has been reported to range from 17-100% [15, 18, 21–35] and from 13-83% in ERα negative breast cancer [17, 24, 29, 30, 33, 36]. With regard to the biological functions of ERβ, a number of studies have shown that the presence of this receptor correlates with improved rates of recurrence, disease-free survival and overall survival [22, 24–27, 37–41] while others indicate little to no correlation [28, 30, 38] or even worse prognosis [33, 42–44]. Lastly, several studies have reported that the presence of ERβ in breast tumors increases the effectiveness of tamoxifen therapy [36, 45–48] or aromatase inhibitor therapy [47, 49]. For these reasons, the expression profiles and biological functions of ERβ in human breast tumors remains unclear and has limited its utility as a prognostic and/or predictive biomarker for this disease. A potential reason for the conflicting data relates to the known existence of at least 4 different ERβ splice variants (ERβ2-5) in human breast tumors whose biological functions largely remain unknown. Additionally, a recent report by our laboratory and others suggests that some of the inconsistencies regarding the prevalence of ERβ in breast tumors may be related to the use of non-specific and/or insensitive ERβ antibodies [20, 50].

For these reasons, we sought to further characterize the expression patterns of ERβ1 across multiple breast cancer sub-types using a validated antibody. This particular antibody (PPG5/10) has been shown by us and others to detect only the full-length form of this receptor and is highly sensitive and specific in immunohistochemical studies [20, 50, 51]. Here, we have examined nuclear and cytoplasmic ERβ1 expression levels in over 400 breast tumors and have correlated these levels with other prognostic biomarkers and/or known patient outcomes. Our results demonstrate that ERβ1 is expressed across all tumor sub-types, including triple negative breast cancers (TNBC), and is significantly associated with improved patient outcomes in women taking tamoxifen for adjuvant therapy of resected, ERα-positive, early stage breast cancer. Based on these observations, we explored the utility of therapeutically targeting ERβ1 using ERβ-specific agonists and multiple anti-estrogenic compounds in both ERα-positive and ERα-negative breast cancers using a number of cell model systems. Our results demonstrate that targeting this receptor results in potent anti-proliferative effects in multiple breast cancer sub-types. However, the effectiveness of these two classes of drugs varies dramatically as a function of ERα status.

## Methods

### Study cohorts

For this study, 3 distinct patient cohorts were utilized to examine the prevalence of ERβ1 expression across multiple breast tumor sub-types and to determine its association with other prognostic biomarkers and response to endocrine therapy. The first cohort (C1) is a retrospectively assembled cohort of 184 women who underwent primary breast cancer surgery at Mayo Clinic Rochester between 2001 and 2008. The second cohort (C2) is a retrospectively assembled cohort of 68 patients who underwent primary breast cancer surgery between 1998 and 2011 at Mayo Clinic Scottsdale, selected for the presence of TNBC on central pathology testing. The third cohort (C3) is a secondary analysis of a prospective adjuvant tamoxifen study in postmenopausal women with early stage ERα-positive breast cancer (North Central Cancer Treatment Group (NCCTG) Trial 89-30-52)) who were randomized to adjuvant treatment with tamoxifen (20 mg per day orally for 5 years) plus fluoxymesterone (10 mg orally twice per day for 1 year) and who had a tumor specimen available from their primary surgery (177 of 258 eligible patients) [52]. All patients enrolled in this study provided informed consent and the use of patient tumor samples for immunohistochemical analysis was approved by the Institutional Review Board at Mayo Clinic (protocol #: 13–000585). Patient characteristics within these three cohorts are shown in Table 1 and the molecular and histologic subtypes represented within each cohort is shown in Table 2.

**Table 1:** **Patient characteristics and clinicopathological variables for each of three cohorts**

Patient characteristics	Cohort 1	Cohort 2	Cohort 3
	n = 184	n = 68	n = 177
median age (range)	58 (28–87)	60 (27–82)	68 (48–89)
Histology			
Ductal	138 (75.0%)	52 (76.5%)	143 (80.8%)
Lobular	28 (15.2%)	0	16 (9.0%)
Other	18 (9.8%)	16 (23.5%)	18 (10.2%)
Receptor status			
ERpos/PRpos or unknown	143 (77.3%)	0	177 (100%)
ERpos/PRneg	27 (14.6%)	0	0
ERneg/PRneg	14 (8.1%)	68 (100%)	0
Her2 status			
positive	27 (14.7%)	0	15 (8.5%)
negative	145 (78.8%)	68 (100%)	160 (90.4%)
unknown	12 (6.5%)	0	2 (1.1%)
Max tumor dimension			
0.1-2.0 cm	115 (62.5%)	42 (61.8%)	
2.1-5.0 cm	51 (27.6%)	21 (30.9%)	
5.1+ cm	18 (9.7%)	5 (7.4%)	*
Number of positive nodes			
0	112 (60.9%)	49 (73.5%)	110 (62.1%)
1-3	46 (25.0%)	14 (20.6%)	47 (26.6%)
4-9	16 (8.7%)	3 (4.4%)	13 (7.3%)
10+	10 (5.4%)	1 (1.5%)	7 (4.0%)
unknown	0	1 (1.5%)	0
Nuclear Grade 3	45 (24.5%)	55 (80.9%)	41 (23.2%)
max Ki67 across all cores			
not done	3 (1.6%)	6 (8.7%)	177 (100%)
0 – 10%	61 (33.2%)	16 (23.2%)	
10.1 – 25%	59 (32.1%)	9 (13.0%)	
25.1 – 50%	40 (21.7%)	6 (8.7%)	
50.1-100%	21 (11.4%)	32 (46.4%)	
ERβ1 nuclear expression			
negative/low (0–2)	121 (65.7%)	51 (75.0%)	32 (18.1%)
moderate (3–5)	59 (32.1%)	17 (25.0%)	96 (54.2%)
high (6–7)	4 (2.2%)	0	49 (27.7%)
ERβ1 cytoplasmic expression			
negative/low (0–2)	164 (89.1%)	45 (66.2%)	1 (0.6%)
moderate (3–5)	20 (10.9%)	21 (30.9%)	52 (29.4%)
high (6–7)	0	2 (2.9%)	124 (70.1%)

*tumor size collected as < 3 m vs. ≥ 3 cm: 140 (79.1%) vs. 37 (20.5%).

**Table 2:** **ERβ1 expression levels by morphology and subtype**

	ERβ1 expression	Cohort 1 n = 184**		Cohort 2 n = 68		n = 177	
		Nucleus	Cytoplasm	Nucleus	Cytoplasm	Nucleus	Cytoplasm
**Molecular Subtype**							
Luminal A (ERα +/ HER2 -/ Ki67 ≤ 10)	Neg/low	31 (18.3)	47 (25.5)				
	Moderate	18 (10.7)	3 (1.6)				
	High	1 (0.6)	0				
Luminal B (ERα +/ HER2 -/ Ki67 > 10)	Neg/low	59 (34.9)	76 (41.3)				
	Moderate	25 (14.8)	11 (6.0)				
	High	3 (1.8)	0				
Her2+	Neg/low	20 (11,8)	24 (13.0)				
	Moderate	7 (4.1)	3 (1.6)				
	High	0	0				
Triple Negative (ERα -/PR-/ HER2 -)	Neg/low	1 (0.6)	5 (2.7)	51 (75.0)	45 (66.2)		
	Moderate	4 (2.4)	0	17 (25.0)	21 (30.9)		
	High	0	0	0	2 (2.9)		
**Histologic Subtype**							
Ductal	Neg/low	93 (50.5)	120 (65.2)	39 (57.4)	34 (50.0)	28 (15.8)	0
	Moderate	42 (22.8)	18 (9.8)	13 (19.1)	16 (23.5)	79 (44.6)	45 (25.4)
	High	3 (1.6)	0	0	2 (2.9)	36 (20.3)	98 (55.4)
Lobular	Neg/low	18 (9.8)	26 (14.1)	0	0	1 (0.6)	0
	Moderate	9 (4.9)	2 (1.1)	0	0	10 (5.7)	3 (1.7)
	High	1 (0.5)	0	0	0	5 (2.8)	13 (7.3)
Other	Neg/low	10 (5.4)	18 (9.8)	12 (17.6)	11 (16.2)	3 (1.7)	1 (0.6)
	Moderate	8 (4.4)	0	4 (5.9)	5 (7.4)	7 (4.0)	4 (2.3)
	High	0	0	0	0	8 (4.5)	13 (7.3)

*ki67 not performed **unable to determine molecular subtype in 15 Cohort 1 pts.

### Tissue microarrays and IHC testing of patient samples

**Figure 1:**
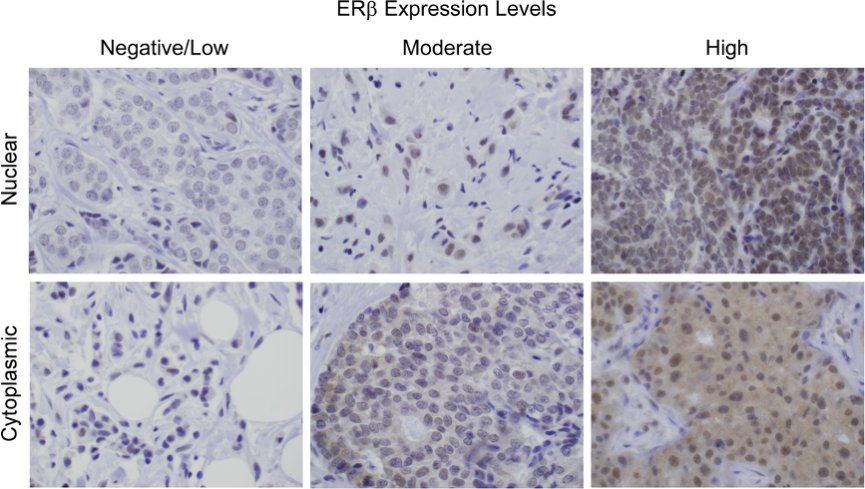
**Immunohistochemical staining for ERβ1 in human breast tumors.** Representative images depicting tumors with negative/low, moderate or high expression of nuclear and cytoplasmic ERβ1 as detected using the PPG5/10 antibody.

Tissue microarrays (TMAs) were constructed for cohorts C1 and C2 using three 0.6 mm tissue cores collected from areas of invasive breast cancer on each tissue block. Five micron sections were cut for immunostaining and analysis as previously described [20]. Full tumor sections from cohort C3 were processed in an identical manner. For HER2 staining, the HercepTest kit (Dako, Carpinteria, CA) was utilized following the manufacturers protocol. All other IHC stains were performed on a Leica Bond III stainer using the following antibodies: 1) A monoclonal ERβ1 PPG5/10 antibody; 1:75 dilution (Thermo Scientific, Waltham, MA), 2) a monoclonal ERα 1D5 antibody; 1:300 dilution (Dako, Carpinteria, CA), 3) a monoclonal PgR 636 antibody; 1:800 dilution (Dako) and 4) a monoclonal Ki67 MIB-1 antibody; 1:300 dilution (Dako). ERα and PgR positivity was determined using standard procedures. Ki67 was scored as previously described [53]. The monoclonal ERβ1 antibody used in this study has been shown to be highly specific and sensitive for detection of only the full-length form of this receptor in IHC studies [20, 50, 51]. Specifically, we have utilized multiple cell model systems which either transiently express ERβ1, or stably express this receptor under the control of a doxycycline inducible promoter, to fully characterize the detection methods and optimal dilution of the PPG5/10 antibody for IHC purposes [20]. Additionally, we have shown that this antibody does not cross-react with ERα or any of the ERβ splice variant forms [20]. Finally, this antibody was compared to multiple other commercially available ERβ specific antibodies and was shown to be one of the best for use in IHC studies using human breast tissue [50]. All slides were reviewed by a dedicated breast cancer pathologist and ERβ1 protein levels were evaluated in both nuclei and cytoplasm. Pathological categorization of ERβ1 levels was determined as a sum of the extent and intensity scores. The extent of staining was scored as follows: 0: less than 1% positive cells, 1: 1%-25%, 2: 26%-50%, 3: 51%-75% and 4: 76%-100%. Intensity of staining was scored as none (0), weak (1), moderate (2) or strong (3). The resulting scores were grouped into 3 categories, namely, ERβ1-negative/low (0–2), ERβ1-moderate (3–5) and ERβ1-high (6–7) and the percentage of tumors falling into these three groups for both nuclear and cytoplasmic staining are indicated throughout this manuscript. A representative tumor determined to be ERβ1-negative, moderate and high is shown in Figure 1 for both nuclear and cytoplasmic localization.

### Cell culture, chemicals and reagents

Parental and ERβ1-expressing MCF7 cells [12] and doxycycline-inducible Hs578T-ERβ1 cells [8] were cultured as previously described. Doxycycline-inducible ERβ1-expressing MDA-MB-231 cell lines were established using the T-REx™ System (Invitrogen) as previously described [9] and were maintained in DMEM/F12 medium supplemented with 10% FBS, 1% AA, 5 mg/L blasticidin S and 500 mg/L zeocin. Charcoal-stripped fetal bovine serum (CS-FBS) was purchased from Gemini Bio-Products (West Sacramento, CA). 17β-estradiol (E2), (Z)-tamoxifen, (Z)-4-hydroxy-tamoxifen and doxycycline (Dox) were purchased from Sigma-Aldrich (St. Louis, MO). (Z)-endoxifen was synthesized by the National Cancer Institute (Bethesda, MD). The ERβ-specific agonists; DPN, WAY200070, FERb 033 and Liquiritigenin, as well as the pure ER antagonist ICI 182,780, were purchased from Tocris Bioscience (Bristol, United Kingdom).

### Real-time reverse transcription polymerase chain reaction

To confirm stable integration and doxycycline inducibility of ERβ1 in the MDA-MB-231 clonal cell lines, cells were plated in 6-well tissue culture plates in the presence and absence of doxycycline (0.1 μg/ml). Following 24 hours of culture, total RNA was isolated using Trizol reagent (Invitrogen), cDNA was synthesized and real-time PCR using ERβ specific primers was performed as previously described [54] and two clones (#4 and 12) exhibiting substantial expression of ERβ1 were chosen for further analysis. To confirm functionality of ERβ1, cells were plated as described above using phenol red-free 10% CS-FBS containing media and treated with ethanol or estradiol (1nM) for 24 hours. RT-PCR was performed using primers specific for the progesterone receptor (PR), PS2 and KLF10 as previously described [12].

### Western blotting

MDA-MB-231-ERβ1 cell lines #4 and #12 were plated in 6-well plates in the presence and absence of doxycycline for 24 hours. Cell lysates were harvested using NETN buffer (150 mM NaCl, 1 mM EDTA, 20 mM Tris [pH 8.0], 0.5% Nonidet P-40), protein concentrations were determined using Bradford Reagent and western blots were performed using Flag (M2, Sigma-Aldrich) and α-Tubulin (DM 1A, Sigma-Aldrich) specific antibodies as previously described [12].

### Proliferation assays

In order to assess anchorage dependent cell proliferation, a crystal violet assay was utilized. This method is well accepted to be reflective of cell number and does not rely on measurements related to mitochondrial activity or intracellular ATP levels that could be compromised by treatments targeting ERβ which is known to be expressed in mitochondria [55–59]. Briefly, cells were plated in replicates of 8 at a density of 1000 cells per well in 96-well tissue culture plates using 10% CS-FBS containing phenol red-free medium. Twenty-four hours after plating, cells were treated with indicated ligands. Cell culture media was replaced every 3 days and crystal violet staining was performed following 12 days of treatment. Crystal violet staining was quantitated using a plate reader set at a wavelength of 550 nm and replicates were averaged among treatment groups.

### Statistical analyses

Descriptive statistics were used to summarize nuclear and cytoplasmic ERβ1 expression levels in each patient cohort. The primary outcome of interest was the recurrence-free interval defined as the time from randomization to documentation of a local, regional, or distant breast recurrence. A stratified log-rank test with strata defined by whether tumor size was ≥ 3 cm and lymph nodes were positive for disease was used to determine whether the recurrence-free interval differed with respect to nuclear or cytoplasmic ERβ1 expression. For all real-time PCR and proliferation assays, a two-sided Student’s *t*-test was utilized. P-values < 0.05 were considered to be statistically significant.

## Results

### Association of ERβ1 with other prognostic biomarkers and tumor grade in an unselected patient cohort

In a cohort of 184 women with primary breast cancer (C1), nuclear ERβ1 expression was determined to be low/negative in 121 (65.7%), moderate in 59 (32.1%) and high in 4 (2.2%) women (Table 1). This is in contrast to cytoplasmic ERβ1 expression that was low/negative in 164 (89.1%) and moderate in 20 (10.9%) women with no tumor exhibiting high cytoplasmic expression (Table 1). The concordance between nuclear and cytoplasmic ERβ1 expression was 66.3% (122/184). ERβ1 was detected across all molecular and histologic subtypes of breast cancer within this patient cohort (Table 2). Moderate to high levels of nuclear ERβ1 expression were detected in 56 of the 170 (32.9%) ERα-positive cases and 7 of the 14 (50.0%) ERα-negative cases (Table 3). In contrast, cytoplasmic ERβ1 expression was similar between the ERα-positive and ERα-negative cancers with approximately 10% of these tumors having moderate to high expression (Table 3). The distributions of nuclear and cytoplasmic ERβ1 expression were similar between HER2 positive and negative tumors; Ki67 low and high tumors; high and low grade tumors; and cases with node positive or negative disease (Table 3).

**Table 3:** **ERβ1 expression levels in a population of breast cancer patients diagnosed at Mayo Clinic Rochester (cohort 1) and its association with other biomarkers, tumor grade and nodal status**

Disease characteristics		ERβ1 status	Nucleus	Cytoplasm
			# of Pts. (%)	# of Pts. (%)
**ERα**	**Positive (n = 170)**	Negative/Low	114 (67.1)	151 (88.8)
		Moderate	52 (30.6)	19 (11.2)
		High	4 (2.4)	0 (0)
	**Negative (n = 14)**	Negative/Low	7 (50.0)	13 (92.9)
		Moderate	7 (50.0)	1 (7.1)
		High	0 (0)	0 (0)
**HER2**	**Positive (n = 27)**	Negative/Low	20 (74.1)	24 (88.9)
		Moderate	7 (25.9)	3 (11.1)
		High	0 (0)	0 (0)
	**Negative (n = 145)**	Negative/Low	94 (64.8)	131 (90.4)
		Moderate	47 (32.4)	14 (9.7)
		High	4 (2.8)	0
**Ki67**	**≤ 10% (61)**	Negative/Low	39 (63.9)	57 (93.4)
		Moderate	21 (34.4)	4 (6.6)
		High	1 (1.6)	0 (0)
	**> 10% (120)**	Negative/Low	79 (65.8)	104 (86.7)
		Moderate	38 (31.7)	16 (13.3)
		High	3 (2.5)	0 (0)
**Tumor grade**	**Grade 1–2 (136)**	Negative/Low	89 (65.4)	122 (89.7)
		Moderate	44 (32.4)	14 (10.3)
		High	3 (2.2)	0
	**Grade 3 (45)**	Negative/Low	31 (68.9)	41 (91.1)
		Moderate	13 (28.9)	4 (8.9)
		High	1 (2.2)	0 (0)
**Nodal disease**	**Present (72)**	Negative/Low	44 (61.1)	65 (90.3)
		Moderate	24 (33.3)	7 (9.7)
		High	4 (5.6)	0 (0)
	**Not present (112)**	Negative/Low	77 (68.8)	99 (88.4)
		Moderate	35 (31.3)	13 (11.6)
		High	0 (0)	0 (0)

### Expression of ERβ1 in triple negative breast cancers

Due to the low number of ERα-negative tumors in our unselected patient cohort (C1), we leveraged another cohort of 68 cases (C2) with confirmed primary TNBC. Nuclear ERβ1 expression was determined to be low/negative in 51 (75.0%) and moderate in 17 (25.0%) tumors (Table 1). This is similar to cytoplasmic ERβ1 expression that was low/negative in 45 (66.2%), moderate in 21 (30.9%) and high in 2 (2.9%) tumors (Table 1). The concordance between nuclear and cytoplasmic ERβ1 expression was 70.6% (48/68). Ki67 results were available in 63 cases. Among the 16 cases whose Ki67 level was not elevated (≤10%), 1 case had moderate levels of both nuclear and cytoplasmic ERβ1 a second case had moderate nuclear expression but negative/low cytoplasmic expression (Table 4). The remaining 14 cases with low Ki67 levels had negative/low nuclear and cytoplasmic ERβ1 expression (Table 4). In contrast, 25 (54.3%) of the 46 cases with elevated Ki67 levels had moderate to high ERβ1 expression in the nucleus and/or cytoplasm (Table 4).

**Table 4:** **ERβ1 expression levels in triple negative breast tumors and its association with Ki67 expression levels**

		ERβ1 Status cytoplasm		
**Ki67 expression**				
	**ERβ1 Status**	Negative/Low	Moderate	High
	**Nucleus**			
**Ki67 > 10% (46)**	Negative/Low	21 (45.6%)	11 (23.9%)	0
	Moderate	4 (8.7%)	8 (17.4%)	2 (4.4%)
	High	0	0	0
**Ki67 ≤ 10% (16)**	Negative/Low	14 (87.5%)	0	0
	Moderate	1 (6.3%)	1 (6.3%)	0
	High	0	0	0

### ERβ and outcomes with adjuvant endocrine therapy

**Figure 2:**
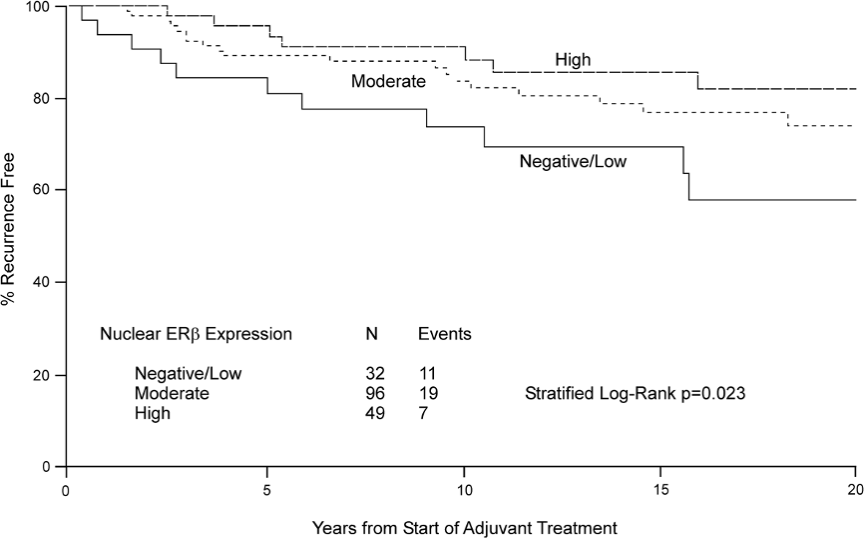
**Increased nuclear ERβ1 expression is associated with prolonged recurrence-free interval in women treated with adjuvant tamoxifen and fluoxymesterone therapy.** Kaplan-Meier estimates of breast cancer recurrence-free interval in patients with negative/low, moderate or high nuclear expression of ERβ1 are shown.

A cohort of 177 postmenopausal women with early stage ERα-positive breast cancer enrolled onto NCCTG 89-30-52 who were randomized to the adjuvant treatment with tamoxifen plus fluoxymesterone arm (C3) was used to assess whether ERβ1 expression is associated with the likelihood of a breast cancer event (local, regional or distant recurrence). With a median length of follow-up of 19.5 years, 56 women are currently alive without disease recurrence, 11 are alive having had disease recurrence and/or a second primary cancer, 49 have died following disease recurrence and/or a second primary cancer and 61 have died without disease recurrence or a second primary disease. Nuclear ERβ1 expression was determined to be low/negative in 32 (18.1%), moderate in 96 (54.2%) and high in 49 (27.7%) women (Table 1). In contrast, cytoplasmic ERβ1 expression was determined to be low/negative in 1 (0.6%), moderate in 52 (29.3%) and high in 124 (70.1%) women (Table 1). As was the case with the other two cohorts, ERβ1 expression was detected across all histologic subtypes of breast cancer (Table 2). The recurrence-free interval (time to local, regional, distant progression) was found to differ with respect to degree of nuclear ERβ1 expression (stratified log-rank test, adjusted for tumor size and node metastasis p = 0.023) with 10 year recurrence-free rates of 74%, 84%, and 88% for patients whose cancers had negative/low, moderate and high levels of ERβ1, respectively (Figure 2). However, the recurrence-free interval was not found to differ with respect to degree of cytoplasmic ERβ1 expression (stratified log-rank test p = 0.623) with 10 year recurrence-free rates of 82% and 84% for patients whose cancers had moderate and high cytoplasmic expression of ERβ1, respectively (Additional file 1: Figure S1).

### Therapeutic targeting of ERβ1 in ERα positive breast cancer cells

**Figure 3:**
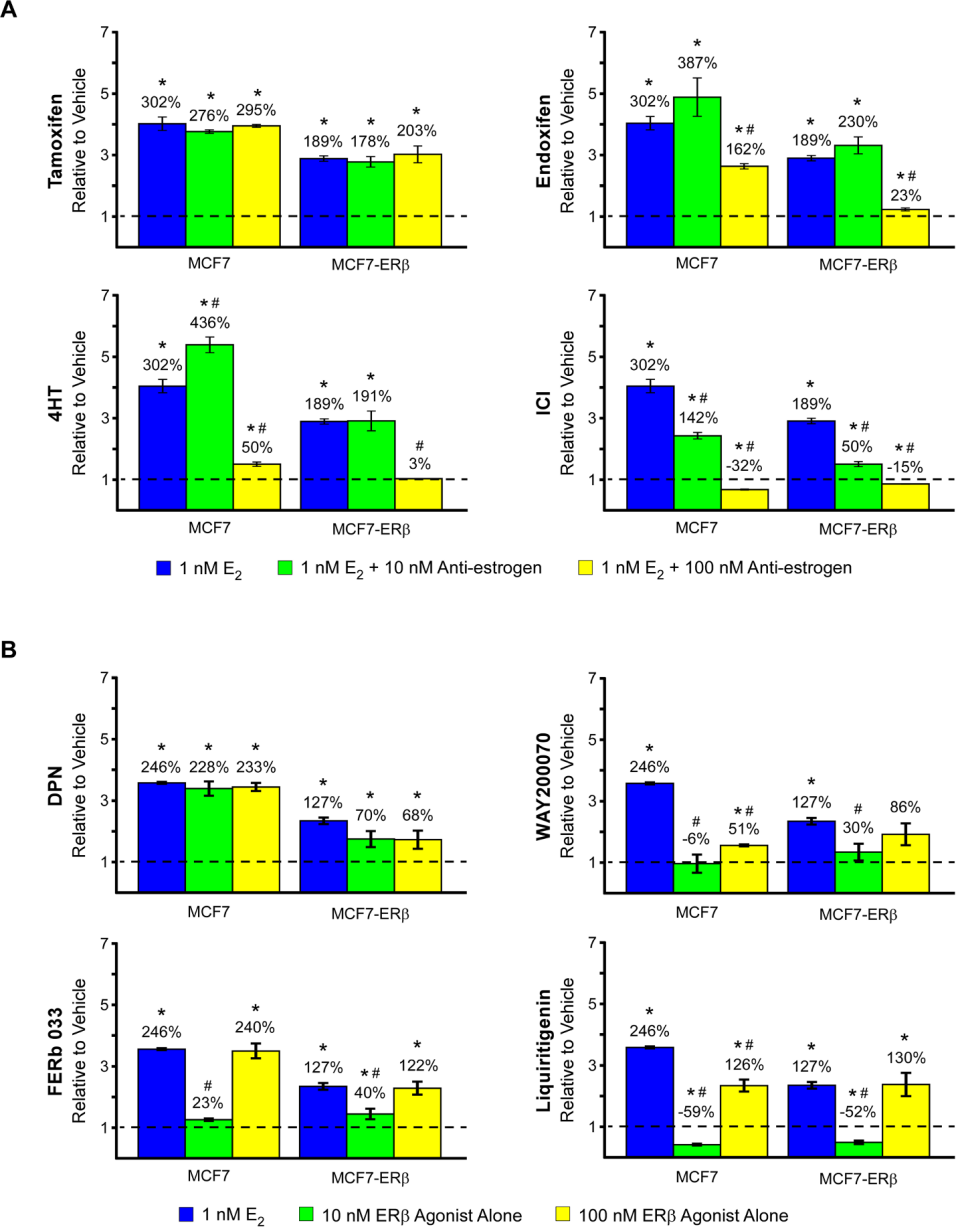
**Effects of anti-estrogenic (A) and ERβ agonist (B) treatment on the proliferation rates of MCF7 and MCF7-ERβ1 expressing cells.** Crystal violet assays were used to determine proliferation rates following indicated treatments for 12 days. P-values < 0.05 were considered to be statistically significant. *Denotes significant difference between indicated treatment and vehicle control treated cells and # between indicated treatment and estrogen treated cells.

Based on the observation that ERβ1 expression is associated with lower rates of recurrence in ERα positive breast cancer, we sought to further characterize the effects of multiple targeted therapies using a breast cancer cell line designed to mimic this tumor sub-type. Therefore, we utilized parental and ERβ1-expressing MCF7 cells previously developed in our laboratory [12]. As a first step, we analyzed the role of ERβ1 in mediating the pro-proliferative effects of 17-beta estradiol (estrogen) and the anti-proliferative effects of anti-estrogenic compounds. As expected, estrogen treatment was shown to induce proliferation in both cell lines; however, the magnitude of induction was decreased in ERβ1 expressing cells (Figure 3A). Tamoxifen had no effect on estrogen-induced proliferation rates regardless of ERβ1 expression (Figure 3A). Interestingly, a low dose (10 nM) of 4HT increased the proliferation rate of parental MCF7 cells above that of estrogen treatment alone, an effect that was not observed in cells expressing ERβ1 (Figure 3A). Higher doses (100 nM) of endoxifen and 4HT, as well as a low dose (10 nM) of ICI, resulted in almost complete blockade of estrogen-induced proliferation in ERβ1-expressing cells but not in parental cells expressing only ERα (Figure 3A).

We next sought to determine if ERβ-specific agonists modulated the proliferation rates of these cells in both the presence and absence of estrogen treatment. In the absence of estrogen (Figure 3B), low (10 nM) and moderate (100 nM) doses of DPN induced proliferation in both parental and ERβ1-expressing MCF7 cells. The magnitude of induction following DPN treatment was nearly identical to that of estrogen treatment in parental MCF7 cells but less than that of estrogen in ERβ1-expressing cells (Figure 3B). Low doses of WAY200070 and FERb 033 had little to no effect on the proliferation rates of parental or ERβ1-expressing cells while higher doses induced proliferation (Figure 3B). A similar pattern was observed following treatment with liquiritigenin with the exception that low doses of this compound were inhibitory regardless of ERβ1 expression (Figure 3B). When each ERβ-specific agonist was administered in the presence of estrogen, the observed dose-dependent effects were abrogated in both cell lines and the proliferation rates of parental and ERβ1-expressing cells were either equivalent or slightly greater than that of estrogen treatment alone (Additional file 2: Figure S2).

### Development and characterization of MDA-MB-231-ERβ1 cell lines

**Figure 4:**
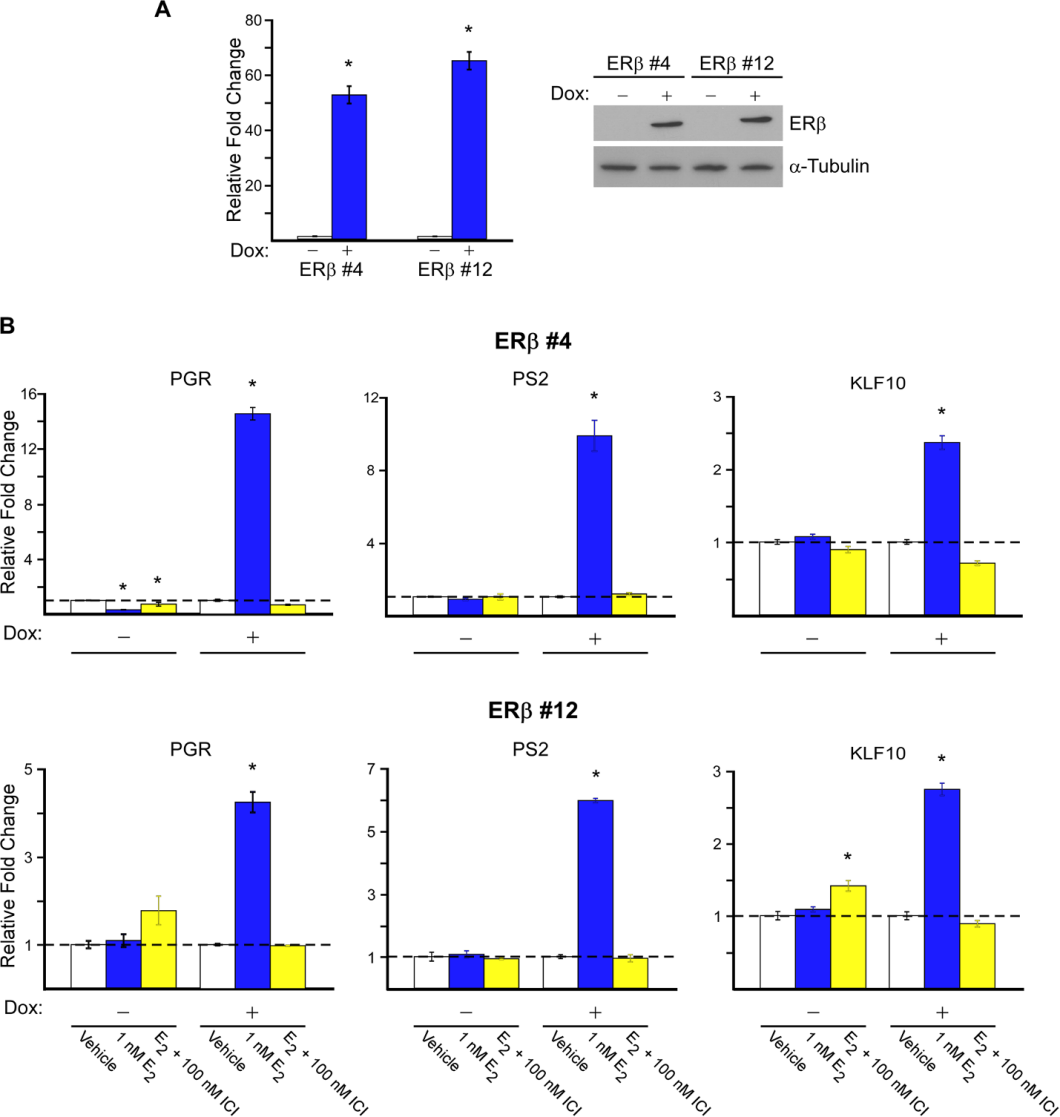
**Characterization of MDA-MB-231-ERβ1 expressing cells. A)**. Real-time PCR and Western blot analysis of MDA-MB-231-ERβ1 clonal cell lines # 4 and 12 indicating mRNA and protein expression levels of ERβ1 in the absence and presence of doxycycline (Dox). **B)**. Real-time PCR analysis of the progesterone receptor (PR), trefoil factor 1 (PS2) and Kruppel like factor 10 (KLF10) following indicated treatments for 24 hours. P-values < 0.05 were considered to be statistically significant. *Denotes significant difference between indicated treatment and vehicle control treated cells.

Since approximately 25% of TNBC were shown to express nuclear ERβ1 (Table 1; cohort 2), we next sought to determine whether expression of ERβ1 in MDA-MB-231 cells, a well-characterized model of TNBC, would alter the cell’s response to ERβ targeting treatments. Two clonal cell lines (#4 and #12) exhibiting robust doxycycline induced expression of ERβ1 mRNA and protein were chosen for further analysis (Figure 4A). To confirm that expression of ERβ1 was exclusively limited to the presence of doxycycline and that the expressed receptor was functional, cells were treated with vehicle, estrogen (1 nM) or estrogen plus ICI (100 nM) for 24 hours and the expression profiles of known ERβ1 target genes were examined. As shown in Figure 4B, these genes were significantly induced following estrogen treatment in the presence of doxycycline, an effect that was completely blocked by the addition of ICI. However, these genes were not induced by estrogen in the absence of doxycycline confirming that these cells do not endogenously express any form of the estrogen receptor (Figure 4B).

### Effects of anti-estrogens and ERβ-specific agonists on the proliferation rates of ERβ1-positive triple negative breast cancer cells

**Figure 5:**
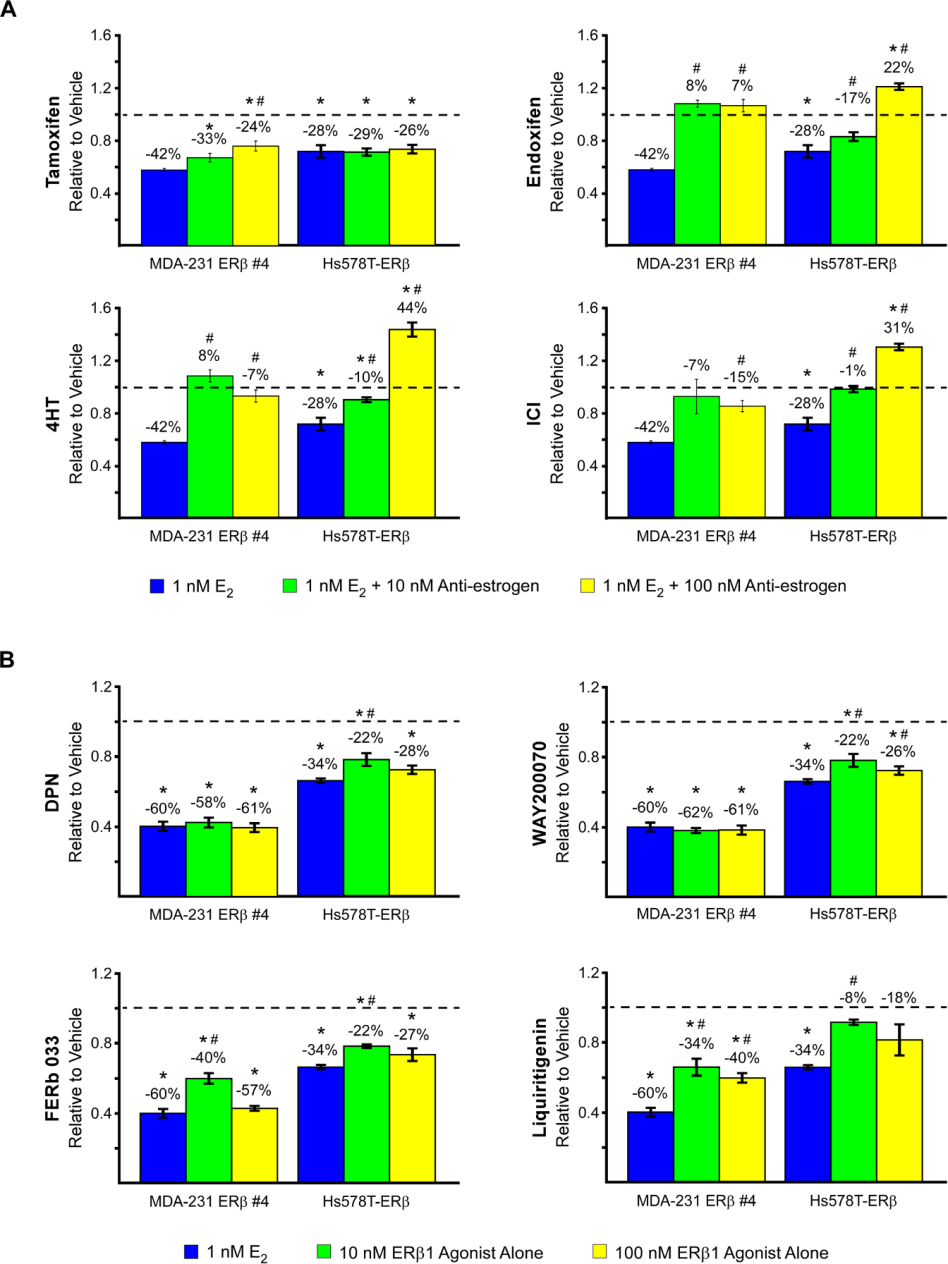
**Effects of anti-estrogenic (A) and ERβ agonist (B) treatment on the proliferation rates of MDA-MB-231-ERβ1 and Hs578t-ERβ1 cells.** Crystal violet assays were used to determine proliferation rates following indicated treatments for 12 days. P-values < 0.05 were considered to be statistically significant. *Denotes significant difference between indicated treatment and vehicle control treated cells and # between indicated treatment and estrogen treated cells.

We next performed a series of proliferation assays to determine which therapeutic strategies may be most effective for the treatment of ERβ1 positive TNBC. Interestingly, estrogen treatment (1 nM) was shown to substantially inhibit the proliferation rates of MDA-MB-231-ERβ1 cells (Figure 5), an effect that was not observed in the absence of doxycycline (data not shown). The addition of multiple anti-estrogens significantly reversed the inhibitory effect of estrogen in MDA-MB-231-ERβ1 cells (Figure 5A). In order to ensure that these effects were not unique to the MD-MB-231 cell line, identical assays were performed using Hs578T-ERβ1 expressing cells [8]. Estrogen treatment significantly repressed proliferation of Hs578T-ERβ1 cells, effects that were reversed following the addition of endoxifen, 4HT or ICI (Figure 5A). Similar responses were observed in the MDA-MB-231-ERβ1 clonal cell line #12 (Additional file 3: Figure S3A).

Since estrogen treatment resulted in substantial reductions in the proliferation rates of ERβ1-expressing TNBC cells, we next analyzed the effects of multiple ERβ-specific agonists in these two cell lines. All of the ERβ-specific agonists tested significantly inhibited the proliferation rates of MDA-MB-231-ERβ1 and Hs578T-ERβ1 cells with DPN and WAY200070 eliciting the greatest responses (Figure 5B). Nearly identical responses were observed in the MDA-MB-231-ERβ1 clonal cell line #12 (Additional file 3: Figure S3B). Combinatorial treatment with 1 nM concentrations of estrogen plus ERβ-specific agonists did not result in greater anti-proliferative effects (data not shown).

## Discussion

In this study, we have compared the nuclear and cytoplasmic expression profiles of ERβ1 across multiple sub-types of breast cancer and in a population of well annotated patients treated with adjuvant endocrine therapy. Our results have revealed that ERβ1 expression, while present in nearly all normal breast epithelium, is lost in many breast cancers. However, the expression of ERβ1 is associated with substantially improved anti-tumor effects in ERα-positive tamoxifen treated breast cancer, as well as potent anti-proliferative effects *in vitro*, confirming its role as a tumor suppressor. Interestingly, the biological effects of therapeutically targeting ERβ appear to be critically correlated with the presence of ERα. In ERα-positive cell lines, expression of ERβ1 enhanced the anti-proliferative effects of anti-estrogenic therapies including endoxifen, 4HT and ICI. However, targeting ERβ with specific agonists in MCF7 cells was not an effective treatment strategy and led to growth stimulation in most instances, likely due to the known cross-reactivity of these compounds with ERα at higher concentrations (100 nM). In contrast, activation of ERβ1 with estrogen or ERβ-specific agonists was shown to substantially repress TNBC cell proliferation rates while the use of anti-estrogens was ineffective and in some cases resulted in stimulation of cell proliferation. Taken together, our studies have comprehensively analyzed the protein expression profiles of ERβ1 across multiple breast cancer sub-types and demonstrated critical roles for this receptor in mediating the effectiveness of multiple therapeutic treatment strategies for breast cancer patients that are related in part to the presence and absence of ERα expression.

Using a well-validated and highly specific antibody and a large cohort of unselected breast cancer patients, we have shown that ERβ1 expression is lost in most cancers as approximately 65% of all breast tumors were determined to be ERβ1-negative. When ERβ1 is expressed, it can exhibit both nuclear and cytoplasmic localization in tumor cells. These data are in agreement with the largest study conducted to date that reported a frequency of 39% for nuclear ERβ1 expression in ERα-positive invasive breast cancers using the same antibody as was used in this study (PPG5/10) [46]. Similar to our findings, this study also did not find an association between ERβ1 expression and other clinicopathological factors [46]. However, it should be noted that others have reported somewhat higher frequencies of ERβ1 positivity using this antibody [26, 29, 30, 45, 60], and one study concluded that ERβ1 was significantly associated with expression of ERα and PR and inversely associated with HER2 overexpression [60]. We also detected ERβ1 expression across all molecular and histologic subtypes of breast cancer. This is in contrast to a recent publication by Huang et al., in which they demonstrated that ERβ1 is only expressed in lobular, and not ductal, carcinomas [21]. These discrepancies may be explained by the use of different antibody dilutions and detection techniques as well as scoring criteria. In alignment with the study by Novelli and colleagues (45), we utilized a conservative approach and categorized tumors exhibiting low expression of ERβ1 as negative.

Although many studies have examined the expression profiles of ERβ1 in ERα-positive tumors, fewer have reported the frequencies of ERβ1 in ERα-negative tumors. To our knowledge, the data reported here are the first to analyze ERβ1 expression in a patient cohort of confirmed TNBC. Of the 68 TNBCs analyzed, 24% expressed nuclear ERβ1 with approximately 34% exhibiting cytoplasmic localization of this full-length receptor. These results are consistent with previous reports, which have suggested that between 24% and 44% of ERα negative tumors, but not necessarily TNBC, are ERβ positive [23, 30, 33, 61, 62]. However, two other publications have reported higher frequencies of ERβ in ERα-negative tumors [32, 63] although it should be noted that in both of these studies, PR and HER2 status were not analyzed and ERα status was determined by ligand binding assays, not immunohistochemistry. None of these studies commented on cytoplasmic expression of ERβ and some studies utilized antibodies that are not specific for full length ERβ and instead can cross-react with its splice variant forms. In contrast to our data in ERα-positive tumors, ERβ1 expression in TNBC was associated with higher expression of Ki67 as has been reported by others [30, 32, 33, 64]. Taken together, these data suggest that the functions of ERβ1 in the absence of ERα expression may be substantially different. Additionally, it is possible that Ki67 levels may vary in ERβ1-positive TNBC based on menopausal status, a possibility that has yet to be examined.

Using a well-annotated cohort of 177 ERα-positive breast cancer patients who were treated with adjuvant tamoxifen (20 mg/day for 5 years) plus fluoxymesterone (10 mg orally twice per day for 1 year), we found that increased expression of nuclear ERβ1 was associated with prolonged recurrence-free interval. To our knowledge, our results are the first prospective-retrospective study of the prognostic value of ERβ1 in patients treated with tamoxifen in the adjuvant setting. These data are consistent with previous studies that have utilized various antibodies specific for full length ERβ1 and have demonstrated that high expression correlates with increased response to tamoxifen therapy [40, 46, 65], improved disease free survival [22, 25–27], longer overall survival [27, 36] and no relapse within 5 years [38]. Although all of the patients included in this cohort were ERα positive, a recent publication has also suggested that ERβ1 may have additional predictive value for tamoxifen responsiveness in ERα negative tumors which express high levels of SRAP [48]. In contrast to prior literature, we also analyzed cytoplasmic staining for ERβ1 and demonstrated no association with the risk of recurrence. While the basis for cytoplasmic localization of ERβ1 is not well understood and remains somewhat controversial, a number of reports have demonstrated that ERβ1 is expressed in mitochondria [55–59] and it is possible that this in part explains the detection of this hormone receptor within this sub-cellular compartment. Interestingly, a recent study has suggested that tamoxifen resistance may develop by agonizing mitochondrial ERβ1 resulting in up-regulation of MnSOD activity and ultimately enhancing cell survival and growth [59]. This could partially explain why cytoplasmic expression of ERβ1 did not correlate with improved tamoxifen responsiveness, as was the case for nuclear ERβ1, in the cohort of patients analyzed here. Additionally, these observations may explain why some studies (which did not distinguish between nuclear and cytoplasmic expression) did not detect an association between ERβ expression and outcomes in tamoxifen treated patients [28] while one study actually reported a non-significant trend towards increased recurrence rates in women with high ERβ expression [30]. Overall, these results suggest that determination of nuclear ERβ1 status will improve our ability to predict an individual’s likelihood of response to adjuvant tamoxifen therapy, effects which may be magnified in the absence of cytoplasmic ERβ1.

Given that ERβ1 is expressed in both ERα-positive and ERα-negative breast tumors, we sought to compare a number of different therapeutic strategies to determine which might be most effective for the treatment of patients with ERβ1-positive breast cancer. Using MCF7-ERβ1 and Hs578T-ERβ1 cell lines previously developed in our laboratory [8, 12], as well as a newly developed MDA-MB-231-ERβ1 cell line whose characterization is described in the present manuscript, we performed cell proliferation assays using anti-estrogenic compounds as well as ERβ-specific agonists. In the ERα-positive MCF7 cell line, ERβ1 expression was shown to diminish the pro-proliferative effects of estrogen, a phenomenon reported previously in ERα-positive cells [66–68]. These results are consistent with the observation that ERα-positive/ERβ1-positive tumors typically have reduced expression of Ki67 relative to ERα-positive/ERβ1-negative tumors. Additionally, ERβ1 expression in these cells enhanced the anti-estrogenic effect of endoxifen, 4HT, and ICI. These data are also consistent with previous reports by our laboratory [12] and others [68–70] demonstrating that ERβ1 expression improves the anti-proliferative effects of 4HT, raloxifene and ICI *in vitro*. These data also correlate with our present studies demonstrating that moderate to high expression of ERβ1 in human breast cancers is associated with improved patient outcomes following tamoxifen therapy.

Treatment of MCF7-ERβ1 cells with 4 different ERβ-specific agonists resulted in variable effects on cell proliferation. Moderate (100 nM) concentrations of all of the ERβ-specific agonists led to induction of cell proliferation. These effects were also observed in the parental MCF7 cell line which does not express ERβ1 and are therefore highly likely to occur through the known activation of ERα at these doses [71, 72]. Low (10 nM) concentrations of these compounds were shown to have minimal effects on cell proliferation rates with the exception liquiritigenin which actually inhibited MCF7-ERβ1 cell proliferation following 12 days of treatment. However, a nearly identical effect was observed in the parental cell line suggesting that ERβ1 is not responsible for mediating this inhibitory effect. Our results are consistent with previous reports demonstrating that low concentrations of WAY200070 and DPN have little to no effect on the proliferation rates of T47D parental or ERβ1 expressing cells [66, 73, 74] while higher concentrations of DPN were shown to stimulate proliferation rates above that of vehicle control treated cells [66]. Taken together, these studies suggest that the use of modern day ERβ-specific agonists for ERβ1-positive tumors is highly unlikely to be beneficial in the treatment of breast tumors which also express ERα. Instead, the utilization of anti-estrogenic therapies is likely to remain a superior choice for this sub-type of breast cancer; at least until more specific and potent ERβ-specific agonists are developed.

In contrast to the results observed in ERα-expressing breast cancer cells, estrogen treatment of two different TNBC lines which were engineered to express ERβ1 led to substantial reductions in cell proliferation rates. These results are consistent with previous reports from our laboratory and others demonstrating that expression of ERβ1 in TNBC cells can lead to suppression of both basal and/or estrogen-mediated proliferation rates [8, 75, 76]. As expected, the addition of anti-estrogens such as endoxifen, 4HT, and ICI blocked these estrogen-mediated effects. However, our present data are the first to demonstrate that the use of ERβ-specific agonists in ERβ1-positive TNBC cells can elicit at least equivalent anti-proliferative effects compared to estrogen treatment alone. As might be expected, combinatorial treatments of estrogen plus ERβ-specific agonists did not result in additive or synergistic effects confirming that these compounds function specifically through ERβ1 in our model systems. Overall, our results indicate that further study of ERβ-targeted therapies is warranted for the treatment of patients with ERβ1-positive TNBC, a subgroup of patients with extremely poor outcomes and for which no form of a targeted cancer therapy is currently available.

## Conclusions

In summary, we have examined the expression patterns of ERβ1 across all sub-types of breast cancer using a highly specific and sensitive monoclonal antibody and have reaffirmed the importance of ERβ1 as a tumor suppressor. Specifically, nuclear expression of ERβ1 is associated with significantly improved outcomes in women treated with adjuvant tamoxifen therapy and these observations were confirmed in cell proliferation assays which demonstrated that ERβ1 expression in ERα-positive MCF7 cells significantly improved their responsiveness to anti-estrogenic therapies. However, activation of ERβ1 with either estrogen or ERβ-specific agonists was shown to result in substantial inhibition of cell proliferation in TNBC cells. These results lay the foundation for future studies aimed at analyzing the anti-tumor activity of ERβ-agonists for the treatment of ERβ1-positive TNBC. The outcomes of such studies could have a dramatic impact on our ability to offer alternative therapies and more effectively treat individuals with this form of the disease.

## Authors’ information

James N. Ingle and John R. Hawse are co-senior authors.

## Electronic supplementary material

Additional file 1: Figure S1: Cytoplasmic expression of ERβ1 is not associated with differences in recurrence-free interval in women treated with adjuvant tamoxifen and fluoxymesterone therapy. Kaplan-Meier estimates of breast cancer recurrence-free interval in patients with moderate or high cytoplasmic expression of ERb are shown. (TIFF 81 KB)

Additional file 2: Figure S2: Effects of ERβ agonist + estrogen treatment on the proliferation rates of MCF7 and MCF7-ERβ1 expressing cells. Crystal violet assays were used to determine proliferation rates following indicated treatments for 12 days. P-values < 0.05 were considered to be statistically significant. *Denotes significant difference between indicated treatment and vehicle control treated cells and #between indicated treatment and estrogen treated cells. (TIFF 603 KB)

Additional file 3: Figure S3: Effects of anti-estrogenic (A) and ERβ agonist (B) treatment on the proliferation rates of MDA-MB-231-ERβ1 cells (clone #12). Crystal violet assays were used to determine proliferation rates following indicated treatments for 12 days. P-values < 0.05 were considered to be statistically significant. *Denotes significant difference between indicated treatment and vehicle control treated cells and #between indicated treatment and estrogen treated cells. (TIFF 805 KB)
