# Supplementary figures and images for: ERβ1: characterization, prognosis, and evaluation of treatment strategies in ERα-positive and -negative breast cancer
Source: BMC Cancer. 2014 Oct 7;14:749. doi: 10.1186/1471-2407-14-749 (PMC4196114; doi:10.1186/1471-2407-14-749)

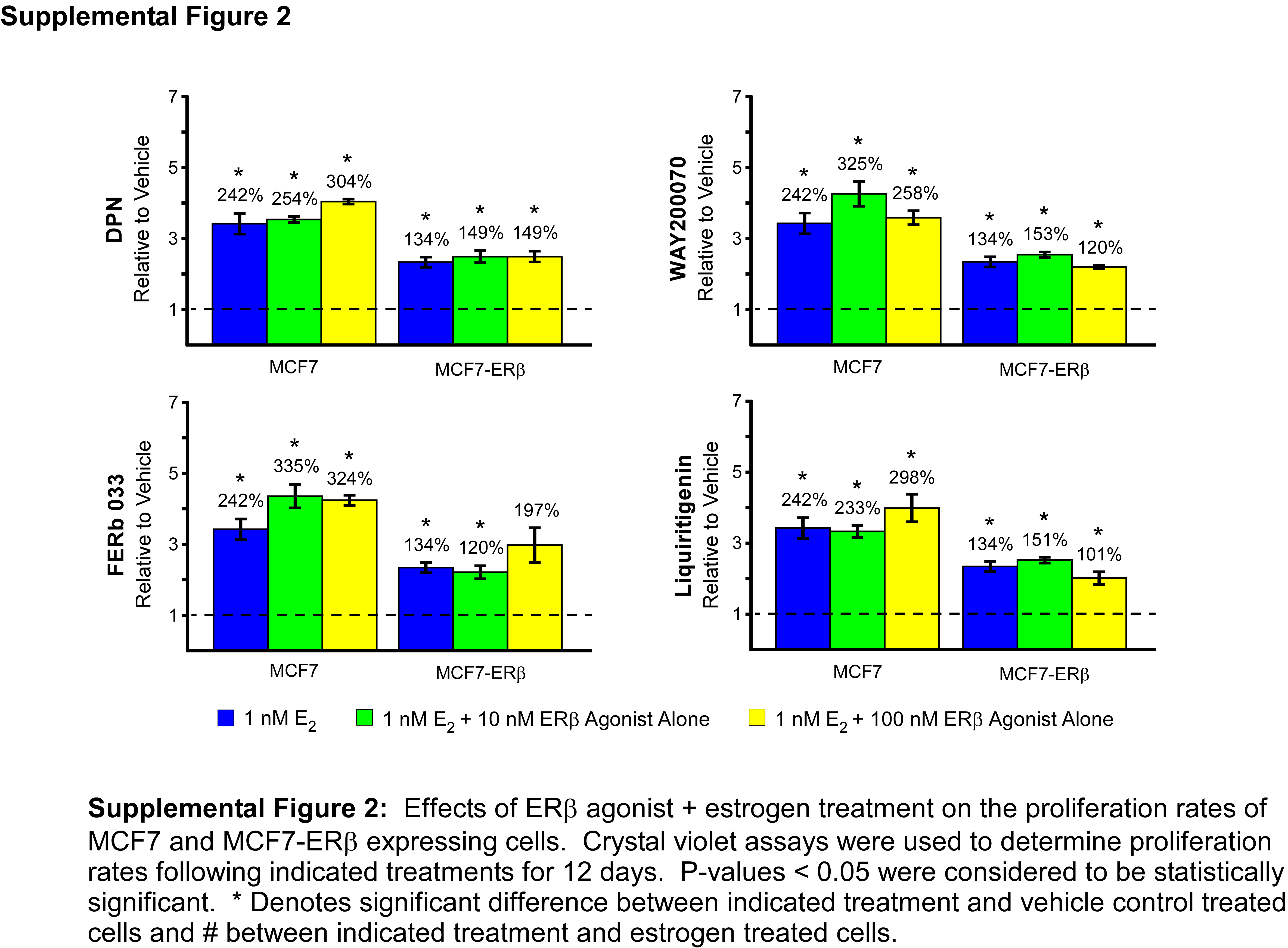

Supplement: Supplementary file 2 — Additional file 2: Figure S2: Effects of ERβ agonist + estrogen treatment on the proliferation rates of MCF7 and MCF7-ERβ1 expressing cells. Crystal violet assays were used to determine proliferation rates following indicated treatments for 12 days. P-values < 0.05 were considered to be statistically significant. *Denotes significant difference between indicated treatment and vehicle control treated cells and #between indicated treatment and estrogen treated cells. (TIFF 603 KB) [file 12885_2014_4927_MOESM2_ESM.tiff]

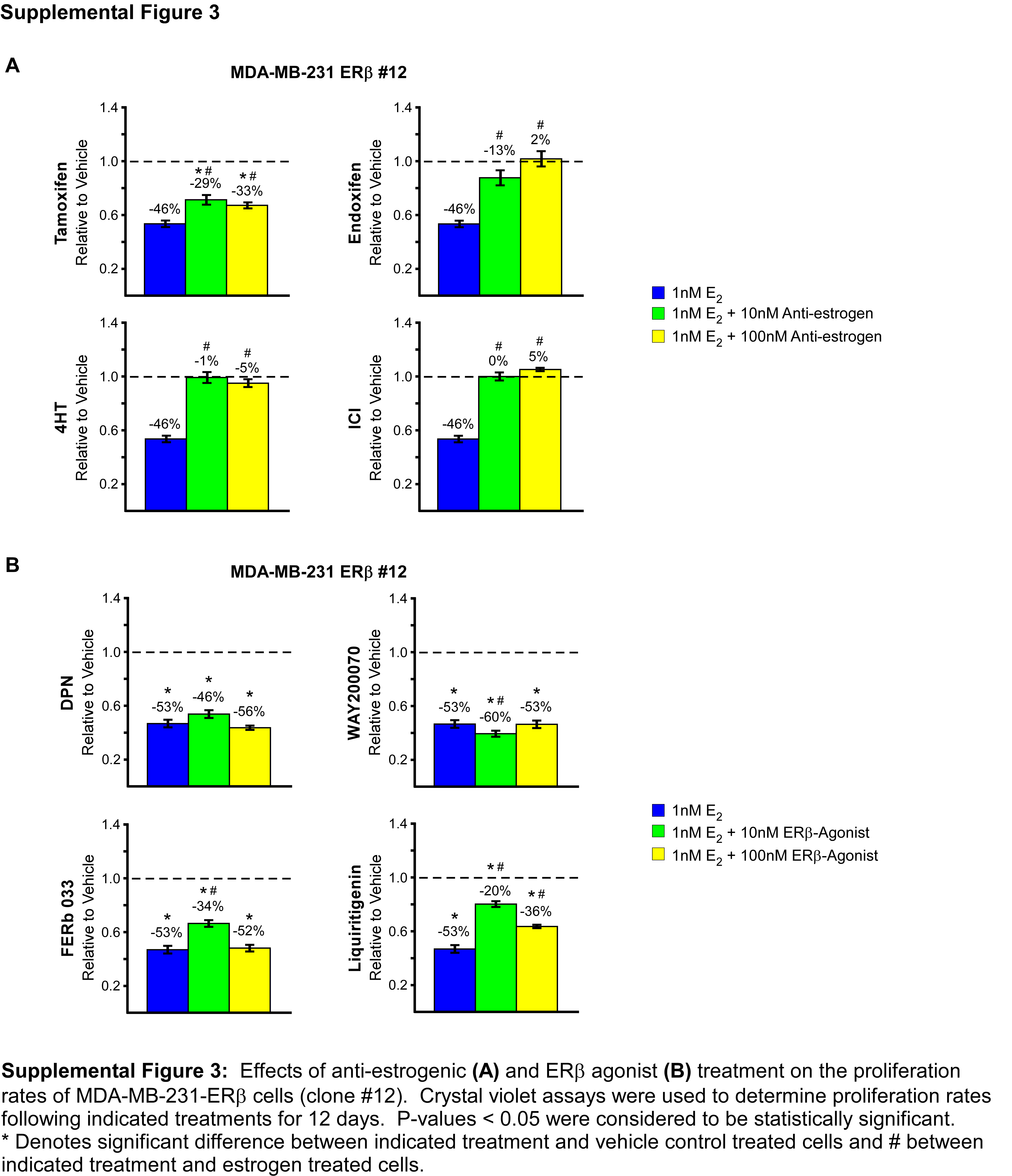

Supplement: Supplementary file 3 — Additional file 3: Figure S3: Effects of anti-estrogenic (A) and ERβ agonist (B) treatment on the proliferation rates of MDA-MB-231-ERβ1 cells (clone #12). Crystal violet assays were used to determine proliferation rates following indicated treatments for 12 days. P-values < 0.05 were considered to be statistically significant. *Denotes significant difference between indicated treatment and vehicle control treated cells and #between indicated treatment and estrogen treated cells. (TIFF 805 KB) [file 12885_2014_4927_MOESM3_ESM.tiff]
